# Insights into the Transport and Thermodynamic Properties of a Bis(fluorosulfonyl)imide-Based Ionic Liquid Electrolyte for Battery Applications

**DOI:** 10.1021/acs.jpclett.1c04246

**Published:** 2022-02-16

**Authors:** Jack Fawdon, Gregory J. Rees, Fabio La Mantia, Mauro Pasta

**Affiliations:** † Department of Materials, University of Oxford, Parks Road, Oxford OX1 3PH, U.K.; ‡ The Faraday Institution Quad One, Harwell Science and Innovation Campus, Didcot OX11 0RA, U.K.; ¶ Universtät Bremen, Energiespeicher-und Energiewandlersysteme, Bibliotechkstraße 1, Bremen 28359, Germany

## Abstract

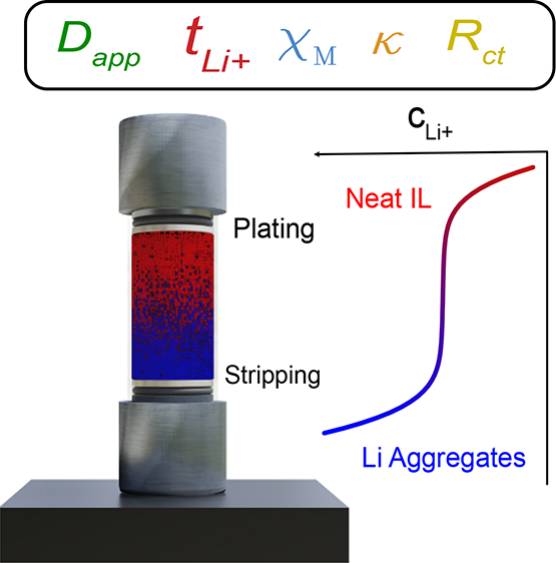

Ionic liquid electrolytes (ILEs) have become popular in various advanced Li-ion battery chemistries because of their high electrochemical and thermal stability and low volatility. However, because of their relatively high viscosity and poor Li^+^ diffusion, it is thought large concentration gradients form, reducing their rate capability. Herein, we utilize operando Raman microspectroscopy to visualize ILE concentration gradients for the first time. Specifically, using lithium bis(fluorosulfonyl)imide (LiFSI) in *N*-propyl-*N*-methylpyrrolidinium FSI, its “apparent” diffusion coefficient, lithium transference number, thermodynamic factor, ionic conductivity, and resistance of charge transfer against lithium metal were isolated. Furthermore, the analysis of these concentration gradients led to insights into the bulk structure of ILEs, which we propose are composed of large, ordered aggregates.

As lithium-ion batteries (LIBs) approach their theoretical energy limit, high-energy alternatives are required for the increasingly high-energy applications society now depends on. Popular strategies to improve energy density include utilizing high-voltage cathodes,^1^ conversion cathodes,^2^ or lithium metal anodes.^3^ Conventional electrolyte compositions used in LIBs, such as 1 M LiPF_6_ in EC:DMC (1:1 v/v), have proven to be unsuitable because of the unfavorable solid (or cathodic) electrolyte interphase (SEI or CEI) that forms.^4^ In recent years, researchers have shown that using ionic liquid electrolytes (ILEs) improves the cyclability because of the stable SEI/CEI on the respective electrode surface.^5−7^ However, with multiple ions in solution and an often high viscosity, ILEs exhibit particularly poor transport properties.^8,9^ This limits their rate performance, as ohmic resistance and concentration gradient formation lead to increasing overpotential with increasing current density. Furthermore, in lithium metal batteries (LMBs), the depletion of Li^+^ at the lithium metal surface has been proven to induce lithium dendrite growth and short-circuiting.^10^

Common ILEs used for battery applications contain 3 or 4 ionic species, and because of the lithium diffusion coefficient  frequently being the lowest and Li^+^ often being present in low concentrations, the transference number of Li^+^ in ILEs has shown to be very low.^11^

The most popular method for determining  in ILEs is via (electrophoretic) pulsed-field gradient nuclear magnetic resonance (pfg-(e)NMR) studies, which explicitly measures the self-diffusion coefficient of each ion in solution (*D*_*i*_). Without an electric field (i.e., pfg-NMR), the transference number can be estimated by calculating the fraction of current carried by Li^+^ using the product of *D*_*i*_ and *c*_*i*_ of each component (partial conductivity). By applying an electric field, pfg-eNMR can measure the mobility of the ions in solution and subsequently isolates the true transference number. These pfg-(e)NMR studies have shown  0.1. Intriguingly, using pfg-eNMR, Gouverneur et al. showed  is negative for LiTFSI in EmimTFSI solution, implying Li^+^ was moving in the “wrong direction”.^12,13^ Others have measured  using electrochemical impedance spectroscopy (EIS) and monitoring the finite-length Warburg diffusion resistance (*W*_d_).^14,15^ However, this method includes an electrolyte ideality assumption, which is especially problematic in a concentrated electrolyte or ILE. To the best of our knowledge, *t*_Li^+^_ values of promising ILEs used in battery applications have not been measured electrochemically via the most defined method of measuring transference, namely, the Hittorf method, nor has the “apparent” diffusion coefficient *D*_app_ been measured via the restricted diffusion method, a common method that measures *D*_app_ by monitoring the semilog decay of open-circuit voltage (OCV) over time, after an arbitrarily formed concentration gradient has been formed.^16−20^ Although the mentioned pfg-NMR and Warburg methods for determining *t*_Li^+^_ are equivalent in “ideal” conditions, using electrochemical methods like Hittorf provides the most rigorous definition of transference in nonideal or concentration electrolytes; therefore, measuring *t*_Li^+^_ via electrochemical means provides the best predictor of performance in nonideal electrolyte conditions. Although the pfg-(e)NMR and EIS techniques have shed some light on the complexities and intricacies of ILE transport, there is yet to be a complete experimental study that monitors both *D*_app_ and , with added thermodynamic understanding provided through a value such as the molar thermodynamic factor (χ_M_).

For a comprehensive understanding of binary electrolyte transport, researchers have utilized operando magnetic resonance imagining (MRI) and Raman techniques to visualize concentration gradients.^21−24^ These studies have not yet been extended to ternary systems or ionic liquid systems. Herein, we use operando Raman microspectroscopy to measure Li^+^ concentration gradients in an IL-based electrolyte system. We focus on 0.5, 1, and 2 m LiFSI in Pyr_1,3_FSI, a three-component, common electrolyte system used in high-energy cells.^5,6,25^ To be explicit, this is the first time ILE concentration gradients have been visualized. Concentration gradient formation is regarded as ILEs’ primary weakness in LIBs and LMBs, so the visualization of the gradient is of particular importance for the understanding and progression of ILEs. Moreover, key electrolyte properties including the “apparent” diffusion coefficient (*D*_app_), lithium transference number (*t*_+_), thermodynamic factor (χ_M_), ionic conductivity (κ), and resistance of charge transfer (*R*_ct_) are isolated. This is also the first time a full suite of electrolyte properties has been measured for a promising ILE for use in battery applications.

*Concentration Gradient Visualization*. Concentration gradients were visualized using operando Raman microspectroscopy (Figure 1), specifically, a time-series of one-dimensional (1D) Raman scans across a custom-built optical Li|Li symmetric cell while current is passed.^22^ Importantly, the cell was placed vertically on the stage, with stripping occurring at the bottom and plating at the top, to avoid natural convection from density differences of the bulk concentration. The line-scan was performed every 4 h for 36 h. Electrolyte solutions were prepared gravimetrically (molal) to increase reliability and accuracy of preparation (for density measurements and molarity equivalents, see Supporting Methods).

**Figure 1:**
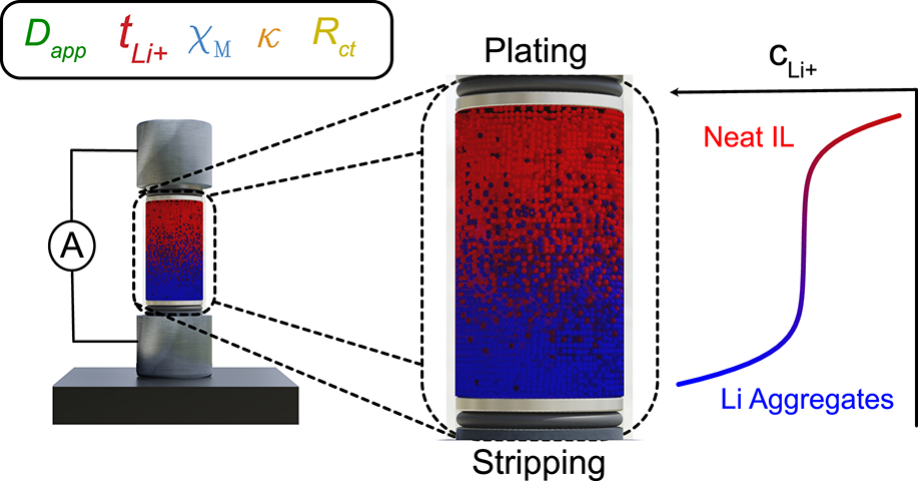
Method for visualizing ILE concentration profile and obtaining *D*_app_, , χ_M_, κ, and *R*_ct_ values. The asymmetry in the concentration gradient is a result of accumulation of Li^+^ species at the bottom of the cell.

*c*_Li^+^_ was calculated by correlating it with the 730 cm^–1^ FSI^–^ peak shift. Representing the S–N–S bending mode,^26^ the 730 cm^–1^ peak shifts to higher wavenumbers monotonically with increasing  because of the continuing formation of high-energy bonding in  structures.^27^ The calibration curve used is shown in Figure 2a, illustrating the nonlinearity of wavenumber increase with  as the LiFSI concentration approaches saturation. An alternative method involved using area ratios FSI^–^ 730 cm^–1^ and  900 cm^–1^ peaks. Because of the increased spectral noise using this method, we selected the former method; further analysis is shown in Supporting Discussion 2.1.

**Figure 2:**
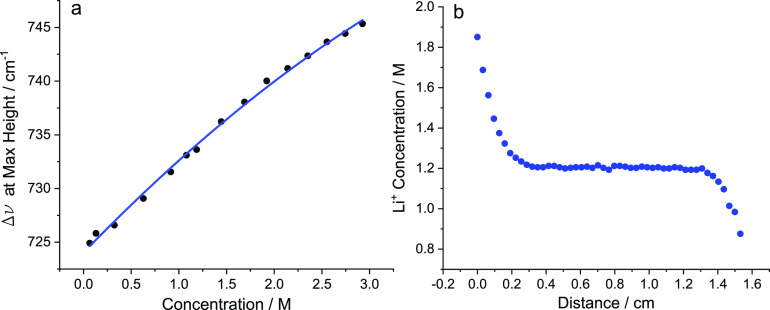
Method for isolating ILE concentration profile: (a) ∼730 cm^–1^ FSI^–^ S–N–S peak shifts to higher wavenumber with Li^+^ concentration. (b) Concentration profile of Li^+^ of 1 m LiFSI in Pyr_1,3_FSI, at 100 μA cm^–2^ after 12 h.

Using the 730 cm^–1^ peak shift method, we checked the mass-balance of the system by integrating each concentration–distance profile, noting if there was any change in the measured profiles over time. Each measurement was within 1.2% of the highest and lowest profile integral over the 36 h experiment. We therefore concluded this is a valid method for calibrating concentration in ILEs.

*Asymmetry and Structural Implications*. Figure 2b shows a concentration profile of Li^+^ in 1 m LiFSI in Pyr_1,3_FSI at 100 μA cm^–2^ after 12 h. Surprisingly, the profile had an asymmetry, with both bulk concentration change and d*c*/d*z* being larger on the stripping side compared to the plating side. This seemed unique to ILE systems, with other systems not showing this phenomenon.^22^

Prior to the application of current, the cell rested for 4 h and a line-scan was recorded. We noticed an increase in concentration at the bottom of the cell, which indicated an accumulation of  before any current was applied (see Figure 3a). This accumulation suggested distinct Li^+^-containing species of higher density were falling because of gravity. To investigate this further, we measured the open-circuit voltage (OCV) of the cell while changing the cell’s orientation. Figure 3b shows how the OCV changed with time, labeled with the orientation of the cell.

**Figure 3:**
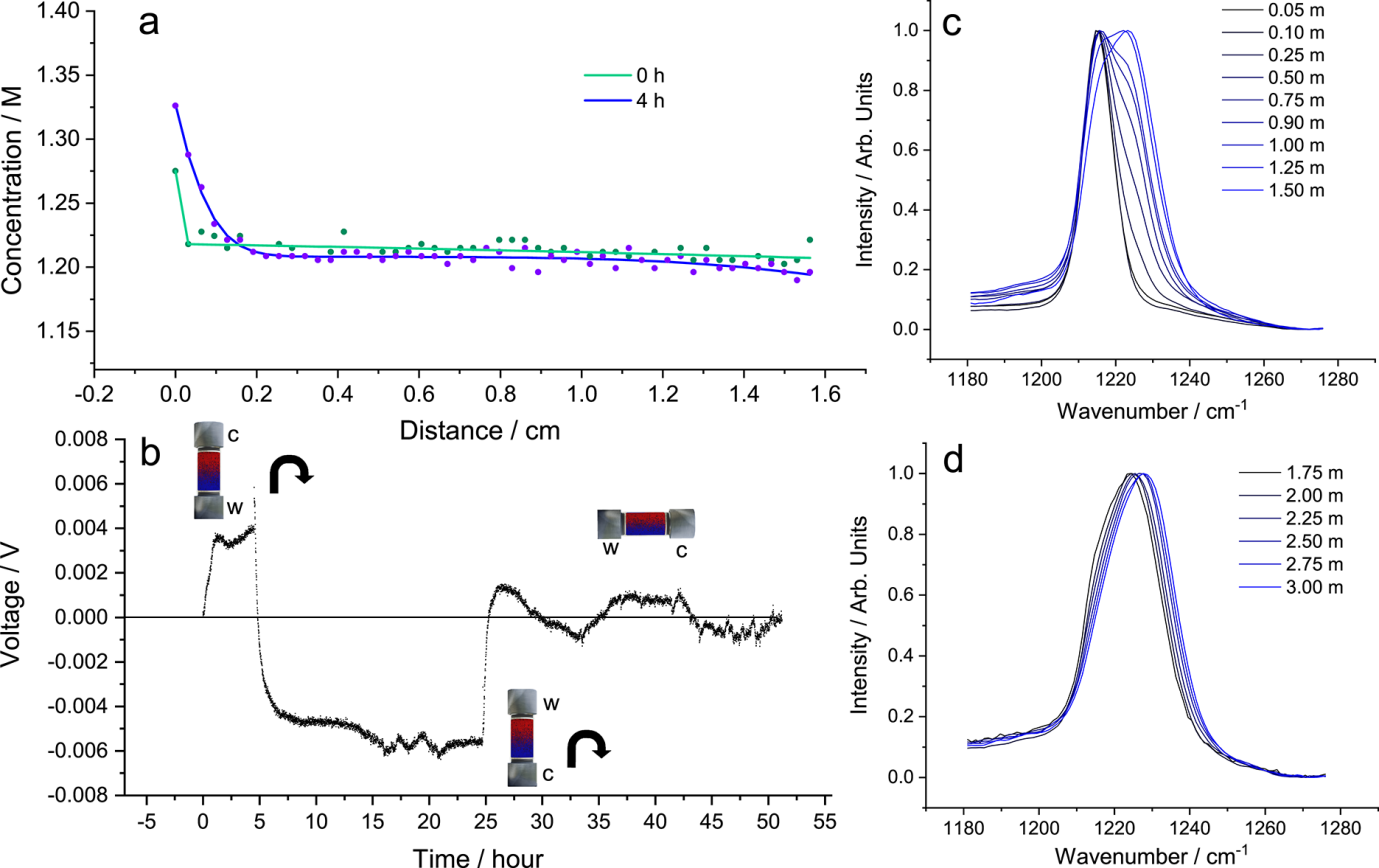
Asymmetric concentration gradients: (a) Formation of concentration differences over 4 h. 0 cm is the bottom of the cell. (b) OCV vs time while no current is passed, showing how orientation affects the OCV of the cell. (c) Raman spectra of 0.05–1.5 m showing two distinct FSI^–^ Raman bands, representing “free” and “bound” FSI. (d) Raman spectra from 1.75 to 3 m showing a peak shift. At ∼1.1 m [FSI^–^] < [Li(FSI)_2_]^−^, which was when the 1225 cm^–1^ became more broad.

It was clear that the OCV was dependent on the orientation of the cell, with it rising from 0 V to ∼4 mV after the first few hours and dropping to approximately −5 mV when the cell was inverted. The cell was then placed horizontally, which led to the OCV reverting back to 0 V. Using 4 mV as the OCV, the thermodynamic activity ratio calculated from the Nernst equation was 1.16, in good agreement with the 1.11 measured by Raman spectroscopy (Figure 3a). As shown in Supporting Figure 3, we also saw this phenomenon with stainless-steel blocking electrodes, albeit to a lesser extent. This suggested that the reactive electrodes increased bulk flow, perhaps because of volume changes caused by the interfacial reaction.^28^

Several molecular dynamic (MD) studies have proposed the formation of long-range ordered structures in ILEs, with some suggesting mesoscopic aggregate formation.^29,30^ Past experimental studies using small-angle X-ray scattering (SAXS) experiments have also predicted the formation of mesoscopic aggregates or domains in neat ILs and with lithium salt in IL solutions.^31−33^ NMR measurements have shown similar results.^34^ However, there is little consensus on the overall size and structure of the aggregates present in IL and Li-salt solutions. Using Stokes’ Law^35^ we estimated the size of aggregates to be 3–8 μm, which is larger than others hypothesized.^31,32^ (See Supporting Discussion 2.2 for further discussion.) However, further studies are required to confirm this value.

The Raman spectra required for  calibration provided information on electrolyte structural changes with increasing LiFSI content. The peak at 1200–1240 cm^–1^ represents the S=O stretching mode of FSI^–^. In the neat IL there was a single peak at 1215 cm^–1^, and with increasing LiFSI addition a new, defined peak appeared at 1225 cm^–1^ (Figure 3c). A defined 1225 cm^–1^ peak is unique to ILEs.^27,36^ This suggested the ILEs have distinctive structures or domains that are not present in organic-based electrolytes. As has been mentioned in other works, the 1215 cm^–1^ peak was speculated to be free FSI^–^ and 1225 cm^–1^ was thought to be a bound Li-FSI_*n*_ species. The solvation number of the Li^+^ can be calculated, as shown in Supporting Discussion 2.4, and we concluded it remained constant at 2 (i.e., Li^+^ is solvated by two FSI^–^, [Li(FSI)_2_]^−^]). With FSI^–^ experiencing two separate environments, it is at ∼1.1 m that [FSI^–^] < [Li(FSI)_2_]^−^. As the concentration increased past 1.25 m, the 1225 cm^–1^ peak became less defined and more broad (Figure 3d), which we speculate could be due to the fusing of the [Li(FSI)_2_]^−^]-derived aggregates forming a homogeneous, percolating network. Indeed, McEldrew et al. predict using MD simulations a “critical threshold” or gelation point where these extended networks form.^37^ Our data agrees with the hypothesis of McEldrew et al., providing experimental evidence to support their claims.

*Li^**+**^ Transport Properties*. The 1 m electrolyte was used as a model system to describe the process of fitting and transport property isolation. Equation 1 is a solution to the diffusion equation in a symmetric cell setup, using the interfacial concentration gradient as a spatial boundary condition.^38,39^ Each gradient was fitted to this equation, elucidating information on the transport properties of the electrolytes. Because of the gradients’ asymmetry, each side of the cell was fitted separately with different diffusion length and interfacial gradient values, with *p* and *s* indicating the plating and stripping sides respectively:
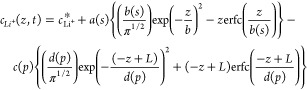
1
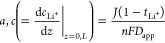
2
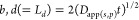
3where  is the concentration of Li^+^ at time *t*, in the vertical *z*-direction;  is the initial lithium concentration; *L* is the interelectrode distance; *J* is the applied current density; *F* is the Faraday constant; *b* and *d* are equal to *L*_*d*_, defined as the diffusion length; and *a* and *c* are equal to , which is the interfacial concentration gradient at each electrode surface, *z* = 0, *L*.

Figure 4a shows  gradients of the 1 m electrolyte at different times. As expected, the gradients were large across the electrolyte, with the stripping electrode showing a significant interfacial concentration (d*c*/d*z*_*z*=0_) gradient of 5.3 ± 0.20 × 10^6^ mol m^–4^ at 100 μA cm^–2^. We also performed the measurement at 50 μA cm^–2^ showing d*c*/d*z*_*z*=0_ as 2.8 ± 0.10 × 10^6^ mol m^–4^. As is expected, d*c*/d*z*_*z*=0_ was directly proportional to the current applied, with d*c*/d*z*_*z*=0_ being almost exactly double when a 100 μA cm^–2^ was applied compared to 50 μA cm^–2^. The plating interfacial gradient (d*c*/d*z*_*z*=*L*_) at 100 μA cm^–2^ was lower at 3.5 ± 0.60 × 10^6^ mol m^–4^, presumably because of the accumulated aggregates at the bottom of the cell.

**Figure 4:**
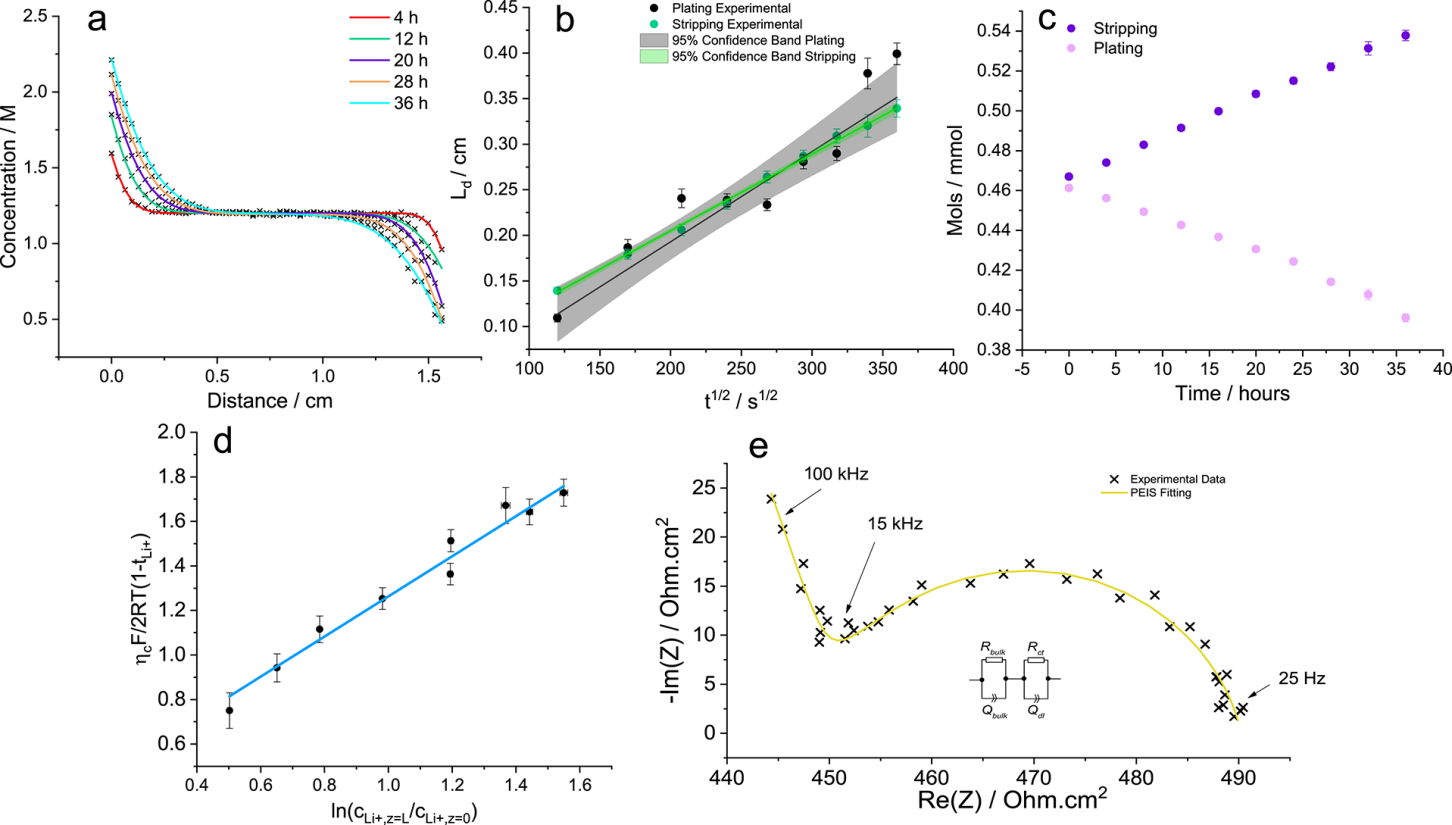
Concentration gradients and the extraction of 1 m LiFSI in Pyr_1,3_FSI electrolyte properties. (a) Li^+^ concentration gradient formation over time, up to 36 h with 8 h gaps. (b) Evolution of diffusion length (*L*_d_) with time on the stripping and plating side. (c) Change in the molar content on the stripping and plating side of the cell with time. (d) Plot of the relationship described by eq 6, illustrating how the η_*c*_ function changes linearly with respect to the natural log of concentration ratio of each cell extreme. (e) Potentiostatic electrochemial impedance spectroscopy (PEIS) of electrolyte prior to application of current, indicating a low *R*_ct_ and *R*_bulk_.

*Diffusion*. By monitoring the diffusion length (*b* and *d*) over time, one can calculate *D*_app_ on both sides of the cell. Figure 4b shows *b* and *d* versus time^1/2^, with the slope being proportional to *D*_app_. Also plotted is the 95% confidence band, which highlights the uncertainty especially on the plating side of the cell. The fitting on the stripping side is much more accurate, which is reflected in the error of the *D*_app_ calculation. On the stripping side, *D*_salt_ = 1.77 ± 0.06 × 10^–11^ m^2^ s^–1^, and on the plating side *D*_app_ = 2.5 ± 0.50 × 10^–11^ m^2^ s^–1^, with an inverse-variance weighted average of 1.78 ± 0.09 × 10^–11^ m^2^ s^–1^. Pulsed field gradient (pfg)-NMR measurements were performed to compare against these values: using the harmonic mean, *D*_salt_ was calculated as 1.77 × 10^–11^ m^2^ s^–1^ (see Supporting Discussion 2.5), very similar to *D*_app_ calculated using concentration visualization. The magnitude of the diffusivities would suggest transport is occurring primarily via an ion-hopping mechanism as opposed to sedimentation of the aggregates identified in the previous sections. However, sedimentation is proposed to be the reason for the asymmetric concentration gradient that was visualized.

*Transference Number*.  was calculated from the fitted concentration gradient. Conventionally,  is measured via the Hittorf method, which looks at calculating the change in concentration on either the plating or stripping side of the cell after a known amount of current is passed.^38^ This was particularly straightforward when utilizing concentration gradient visualization techniques, as one can monitor the concentration on each side of the cell by integrating under the concentration curve. Moreover, those using a conventional Hittorf setup would not notice the initial gradient from the settling aggregates. To the best of our knowledge, the Hittorf method has not been utilized for studying  in lithium-ion room-temperature ILEs, with the majority of groups using pfg-(e)NMR and others describing “ionic melts”.^40−44^ Like Gouverneur, who used pfg-eNMR, we used an “external” reference, namely, the center-of-mass reference.  was calculated:
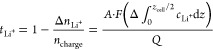
4
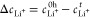
5where *n*_charge_ is the number of moles of charge passed;  is the molar difference between the two sides of the cell before and after time, *t*; *Q* is the charge passed over time; and *A* is the area of the electrode.

Figure 4c shows how the concentration changed for the stripping and plating side; note the change in area is linear, indicating the movement of the aggregated structures remained constant, and so did . Using the initial concentration profile prior to application of current,  was calculated for each scan over time, and the average  was calculated from the inverse-variance weighted mean. On the stripping side  was calculated as −0.088 ± 0.024 and on the plating side, 0.114 ± 0.062, with a weighted average of −0.062 ± 0.070. Again, the lower value on the stripping side was likely due the accumulation of higher-density aggregates on oxidation of Li.

By using the pfg-NMR diffusivities and measured concentrations,  via pfg-NMR was 0.0941, but without an electric field (like in pfg-eNMR), migration was not taken into account.

*Thermodynamic Factor*. χ_M_ correlates the electrolytes’ thermodynamic activity with concentration.^45^ To our knowledge, no room-temperature Li-ion ILEs’ χ_M_ values have been reported, but the activity of various LiNO_3_–AgNO_3_ melt compositions (at 260 *°*C) were measured by Richter a few decades ago.^46^ χ_M_ was calculated as
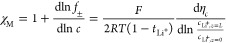
6where *f*_±_ is the molar activity coefficient. Using the potentiostatic electrochemical impedance spectroscopy (PEIS) data prior to each line scan, η_*c*_ was calculated by η_*c*_ = η_total_ – *I*(*R*_bulk_ + *R*_ct_), where η_total_ is measured from the chronopotentiometry data and the resistances are from PEIS. χ_M_ was measured as 0.906 ± 0.064 (Figure 4d), which is reasonable if one were to compare against Richter’s findings, which showed values of between 0.95 and 1.7 as the mole fraction changed between 1 and 0 of AgNO_3_.

*Ionic Conductivity and Resistance of Charge Transfer*. From PEIS, prior to current being passed the ionic conductivity (κ) and resistance of charge transfer (*R*_ct_) were calculated from fitted Nyquist plots (Figure 4e). κ was calculated as 3.52 ± 0.01 mS cm^–1^, which agreed well with previous literature values. Using the pfg-NMR data, the inverse Haven ratio was calculated as 0.520, showing a significant amount of ion–ion correlation. *R*_ct_ was calculated as 44 ± 4 Ω· cm^2^, which is assumed to be a combination of classical charge-transfer and SEI resistance. ILEs containing FSI^–^ in particular have been shown to have fast charge-transfer kinetics, as illustrated by their low *R*_ct_ value.^47,48^

*Dependence on Concentration*. To understand transport changes with a varying amount of Li^+^ present, we performed operando Raman experiments on two other ILE concentrations, namely, 0.5 and 2 m. Like the measurements performed with the 1 m electrolyte, 100 μA cm^–2^ was applied. With 0.5 m, we also performed a measurement at 50 μA cm^–2^ because at the higher current  dropped very quickly at the plating side. Two measurements were run per concentration. Each *D*_app_ and  value can be compared to the pfg-NMR values in Table 1.

**Table 1:** pfg-NMR Diffusivities and Transference in 0.5, 1, and 2 m at 25 *°*C

*c*_Li^+^_ (M)	*c*_FSI^–^_ (M)	*c*_Pyr^+^_ (M)	*D*_Li^+^_ (×10^–11^ m^2^ s^–1^)	*D*_FSI^–^_ (×10^–11^ m^2^ s^–1^)	*D*_Pyr^+^_ (×10^–11^ m^2^ s^–1^)	*D*_salt_ (×10^–11^ m^2^ s^–1^)	*t*_Li^+^_
0.62	4.38	4.08	2.04	2.79	2.47	2.58	0.053
1.19	5.06	3.87	1.42	1.88	1.76	1.77	0.094
2.14	5.63	3.46	0.81	0.97	0.92	0.92	0.169

Figure 5 shows how electrolyte transport and thermodynamic properties were affected by concentration. *D*_app_, , and d*c*/d*z*_*z*=0,*L*_ were taken from the inverse-variance weighted average of the stripping and plating sides. Most strikingly, 0.5 m ILE showed many transport and thermodynamic values similar to those of 1 m. For instance, 0.5 m showed d*c*/d*z*_*z*=0,*L*_ equal to ∼5.50 × 10^6^ mol m^–4^, like that of 1 m.  for both these concentrations was very low, although because of the error involved it is difficult to report whether the values were negative or positive. Values of χ_M_ were lower than 1 for both 0.5 and 1 m, indicating their activity is lower than their concentration because of a high amount of association.

**Figure 5:**
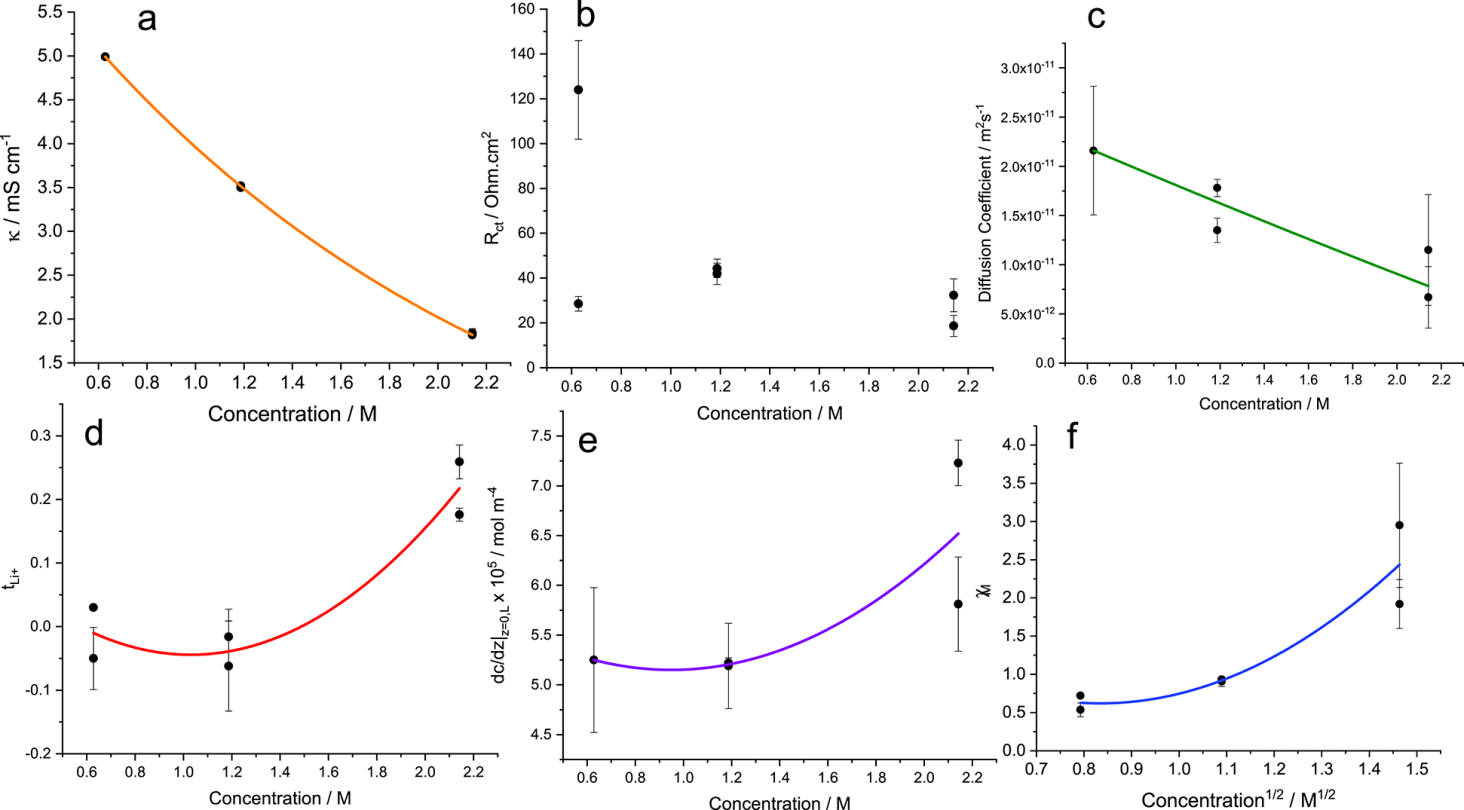
LiFSI in Pyr_1,3_FSI concentration-dependent transport and thermodynamic properties: (a) Ionic conductivity (κ), fitted by exponential decay. (b) Resistance of charge transfer (*R*_ct_) showing a marginal increase at 1 m, then a decrease again at 2 m. (c) Fickian diffusion coefficient (*D*_app_), showing a change moving from 1 to 2 m. (d) Transference number of lithium , initially very low but showing a marked increase from 1 to 2 m. (e) (d*c*/d*z*)_*z*=0_ at 100 μA cm^–2^, which was initially constant but showed an increase moving to 2 m. (f) χ_M_ showing values below 1 for concentrations below 1 m, but increasing to ∼2.5 at 2 m. Values below 1 indicate increased association between Li^+^ and FSI^–^, and values above 1 indicate the decreasing amount of free FSI^–^ present and thus an increase in “effective concentration” of Li^+^. The error bars represent the standard deviation of the inverse-weighted mean of the stripping and plating sides from the fitting of the asymmetric gradient. Experimental inconsistencies such as dendrite formation and small temperature variations explain some differences between the calculated property values for repeated experiments.

For 2 m, there was a noticeable change in many of the transport and thermodynamic properties. For example, there was a marginal increase in d*c*/d*z*_*z*=0,*L*_ from ∼5.5 to ∼6.50 × 10^6^ mol m^–4^ because of changes of *D*_app_ and/or . There was a drop in *D*_app_ versus 1 and 0.5 m. Most certainly there was an increase in , which indicated a structural change perhaps correlated to the broadening of the 1200–1240 cm^–1^ peak. An increase in χ_M_ at high concentrations is common among electrolyte solutions and is noticeable here too. Furthermore, results from Richter showed a similar behavior. We speculate that as more LiFSI was added there were fewer free FSI^–^ to stabilize Li^+^ via extended [Li(FSI)_2_]^−^ structures; χ_M_ then began to rise. There was no noticeable trend in *R*_ct_, with a clear anomaly for one of the 0.5 m samples, which did not appear to affect the other bulk electrolyte property values described.

This reported data suggested there was a transport mechanism change moving from 1 to 2 m. We also speculated above that between these concentrations there was a structural change, as illustrated from the Raman data. We speculate that when [Li(FSI)_2_]^−^ > (FSI)^−^, ordered networks form, increasing .

In summary, by combining spectroscopic and electrochemical techniques with concentration visualization we have presented particularly valuable findings not yet reported in the ILE literature. Specifically, the Li^+^ concentration gradient in ILEs has been visualized for the first time, along with the isolation of key transport and thermodynamic properties. With ILEs’ main weakness being their transport properties, understanding fully the origin of this is paramount for their continuing development. Moreover, this is the first time a thermodynamic understanding of promising battery ILEs has been measured through χ_M_. Additionally, the sedimentation of clustered aggregates have been detected, which has not yet been experimentally measured in the academic literature until now. We anticipate this work to further promote concentration visualization’s unique ability to fully understand electrolyte properties, and specifically, we hope our findings regarding ILEs’ properties and structure will inform their ongoing progress.

## Methods

*Electrolyte Description*. Lithium bis(fluorosulfonyl)imide (LiFSI) (battery grade, 99%) was purchased from Fluorochem Ltd. *N*-Propyl-*N*-methylpyrrolidinium bis(fluorosulfonyl)imide (Pyr_1,3_FSI) (99.9%) was purchased from Solvionic. Handling of LiFSI and Pyr_1,3_FSI was always performed in an argon-filled glovebox (MBraun) with low H_2_O content (<1 ppm) and low O_2_ content (<1 ppm). LiFSI was dried further under high vacuum at 70 °C for 48 h. Pyr_1,3_FSI was dried under high vacuum at 70 °C for 24 h, with a stirrer bar. The H_2_O content of the electrolyte solutions was determined by Karl Fischer titration, which was also performed in an argon-filled glovebox, and recorded to be below 15 ppm of H_2_O.

*Calibration*. Using a confocal Renishaw inVia Reflex laser confocal Raman microscope equipped with a near-IR 785 nm laser, a 5× magnification objective (Leica, 0.12 NA, 14 mm WD), leading to a 4.8 μm spot size, along with a 90*°* mirror (Renishaw) was used to collect Raman spectra of the prepared solution. An even distribution of calibration electrolytes was prepared, between 0.1 and 3 M, inside an Ar-filled glovebox. For each calibration solution, the spectra were recorded with an 800 cm^–1^ center, at 5% laser power, one second exposure, and 20 acquired spectra. Using Renishaw WiRE 5.5 software, the background of each spectrum was removed and normalized. Each 730 cm^–1^ peak was fitted with an exponentially modified Gaussian (EMG) function, and the wavenumber number (*x*-axis) at maximum height was calculated. The calibration curve is shown in Figure 2a.

*Cell Construction*. The custom-designed cell was constructed in an Ar-filled glovebox. Two Li discs of 8 mm diameter were cut and placed onto two stainless steel pistons of the same diameter. One piston was placed into a fused quartz tube; electrolyte was added, and the second piston was added to seal the cell, being careful not to introduce any bubbles into the system. Once sealed, the cell was placed onto the Raman stage vertically and connected to a Biologic SP150 potentiostat.

*Line Scan*. 100 μA cm^–2^ was applied to the cell, and a 1D line scan in the *z*-direction was performed every 4 h for 36 h. The same laser settings used for calibration were used for the line scan too. A point-by-point line scan was taken, with equal spacing between the 1.5 cm interelectrode distance. The confocality of the instrument allowed us to measured the line scan in a precise plane of focus; each measurement was 0.5 cm inside the ID of the quartz tube. Using Renishaw’s WiRE5.5 software, the background was removed, and each spectrum was compared to the calibration using a Python script. The resulting concentration gradient was fitted with eq 1. The Python scripts used in this work are available at github.com/JFawd.

*PEIS*. Before any line scan, potentiostatic electrochemical impedance spectroscopy (PEIS) was performed on the cell. It was also performed between line scans to provide an estimation of η_*c*_. A voltage amplitude (*V*_a_) of 100 mV was used, which allowed for linearity. The frequency was scanned from 100 kHz and 1 Hz. The Nyquist plot was fitted with the equivalent circuit (*Q*_bulk_/*R*_bulk_) + (*Q*_dl_/*R*_ct_), with *R*_bulk_ and *R*_ct_ representing the bulk resistance of the electrolyte and charge transfer, respectively, and *Q*_bulk_ and *Q*_dl_ are the constant phase element of electrolyte and double layer, respectively. Each spectrum was fitted using Biologic EC-lab V11.26 software.

*pfg-NMR*. All pulsed field gradient (PFG) nuclear magnetic resonance (NMR) measurements were completed at 9.45 T (_0_(^1^H) = 400.20, _0_(^19^F) = 376.58, and _0_(^7^Li) = 155.53 MHz) on a Bruker Avance III HD spectrometer using a 5 mm single-axis diffusion probe with exchangeable ^1^H, ^19^F, and ^7^Li ceramic heads. A stimulated echo pulse sequence was utilized with an effective gradient pulse duration (δ) of between 1 and 2 ms, a diffusion time (Δ) of 43–46 ms, with the gradient amplitude varied between 0.1 and 5 T/m. All samples were sealed in a J-Young valve NMR tube; the temperature was stabilized at 298.1 K, and a 5 s recycle delay was used throughout.

## Supporting Information Available

Experimental setup and densitometry measurements; comparison between calibration methods; estimation of aggregate size with Stokes’ Law; description of gradient formation with blocking electrodes; solvation number calculation of Li^+^ using Raman; description of *D*_app_ calculation using pfg-NMR (PDF)
Transparent Peer Review report available (PDF)
