# Supplementary material for: Insights into the Transport and Thermodynamic Properties of a Bis(fluorosulfonyl)imide-Based Ionic Liquid Electrolyte for Battery Applications
Source: J Phys Chem Lett. 2022 Feb 16;13(7):1734–41. doi: 10.1021/acs.jpclett.1c04246 (PMC9084600; doi:10.1021/acs.jpclett.1c04246)
Supplement: Supplementary file 1 — jz1c04246_si_001.pdf [file jz1c04246_si_001.pdf]

Supporting Information

Insights into the Transport and Thermodynamic Properties of a Bis(fluorosulfonyl)imide-based Ionic Liquid Electrolyte for Battery Applications

Jack Fawdon[a], Gregory J. Rees[a,b], Fabio La Mantia[c] and Mauro Pasta[a,b,*]

[a]Department of Materials, University of Oxford, Parks Rd, Oxford, OX1 3PH, U.K.
[b]The Faraday Institution Quad One, Harwell Science and Innovation Campus, Didcot, OX11 0RA, U.K.
[c]Universtät Bremen, Energiespeicher-und Energiewandlersysteme, Bibliotechkstraße 1, Bremen, 28359, Germany
[*]mauro.pasta@materials.ox.ac.uk

1 Supporting Methods

1.1 Densitometry

We used densitometry to convert molality-based concentrations into molarity. We used an Anton Paar DMA 4100 density meter, located in an Argon-filled glovebox. Refer to this work for further details. The density curve is presented in Supporting Figure 1a.

$$c_{molarity} = \frac{n_{mols}\rho}{m_{total}} \tag{1}$$

Supplementary Table 1: *Molality to Molarity*

**Molality / mol kg^{-1}**	**Molarity / mol dm^{-3}**
0.05	0.066
0.50	0.63
1.00	1.19
2.00	2.15
3.00	2.93

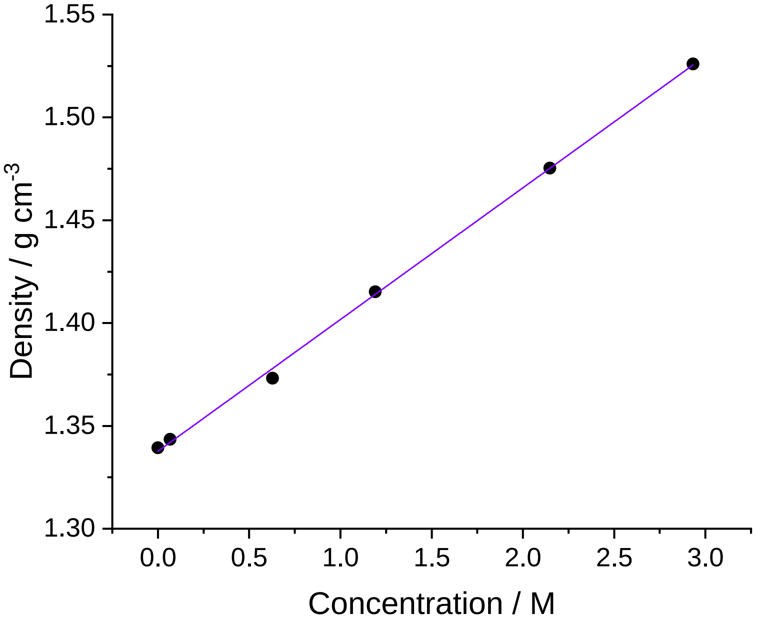

Supporting Figure 1: *Density curve of LiFSI in Pyr*$_{1,3}$ *at 25* ^{o}*C, with a linear fit.*

2 Supporting Discussion

2.1 Calibration Method

As an alternative calibration method we calculated the area ratio of the FSI^- 730 cm^{-1} and $Pyr_{1,3}^+$ 900 cm^{-1} peaks. Supporting Figure 2a the area ratio of the FSI^- 730 cm^{-1} and $Pyr_{1,3}^+$ 900 cm^{-1} peaks' dependency on Li^+ concentration, noting the non-linearity suggesting bulk electrolyte structural changes. An example illustrating each calibration method on a line-scan of a 1 m electrolyte, using a 100 μA cm^{-2} current density after 12 h, is shown in Supporting Figure 2 2b. We note the shape is the same, but using the "area ratio method" led to a much more noisy concentration profile and problems with accuracy of concentration determination from the calibration curve. This was due to the influence the glass-background had on the analysis and mossy-dendrite growth close to the plating surface.

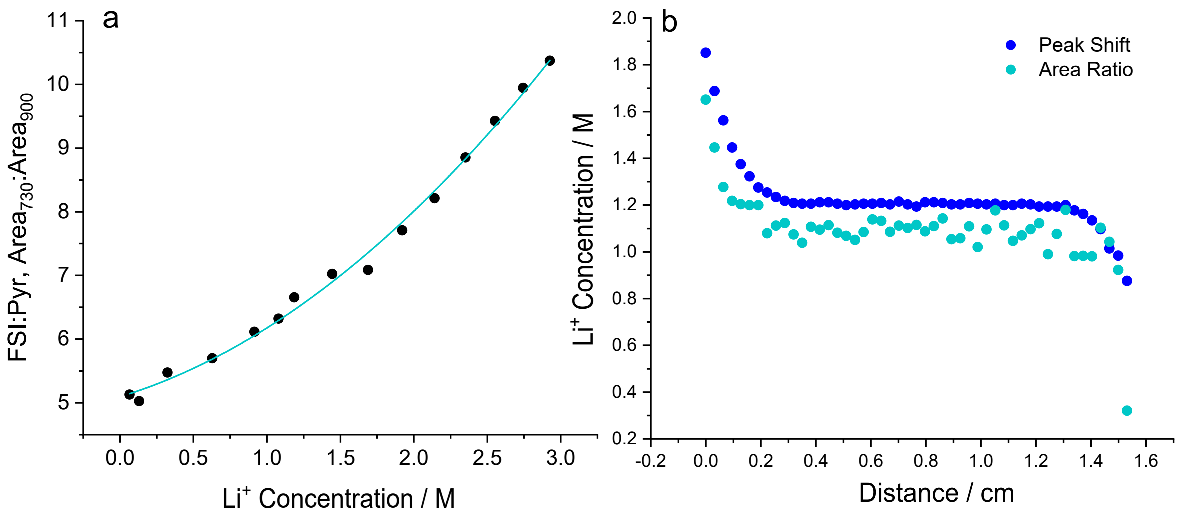

Supporting Figure 2: *Comparison between calibration using wavenumber peak shift of 730 cm*-1 *peak shift and area ratio of of the FSI*$^-$ *730 cm*$^{-1}$ *and Pyr*$^+_{1,3}$ *900 cm*$^{-1}$ *peaks.*

2.2 Stokes' Law

To estimate the size of the Li^+ aggregate structures forming we used Stokes' law,[1] measuring the sedimentation time for the OCV vs. time graph in Figure 3b.

$$v = \frac{2}{9}(\rho_p - \rho_l)\frac{gr^2}{\eta} \quad (2)$$

Where v is the velocity (v = height/sedimentation time), ρ_p is particle density, ρ_l is the viscosity of the liquid, g is the gravitational acceleration (= 9.81 m/s^2), η is the dynamic viscosity and r is the particle radius. A range was estimated for particle size.

The density difference in aggregate and surrounding liquid was estimated, with an upper limit of ρ_{LiFSI} for ρ_p and ρ_l was used as the neat IL. η of the neat IL was measured as 40.7 cP. h was estimated was used as half the distance between the electrodes (7.5 mm). The calculated aggregate size was 3-8 μm. Although the density differences and between neat IL and aggregate, and velocity, were estimated, it can be assumed that the particles are on a micron-scale.

2.3 Blocking Electrode Concentration Gradient

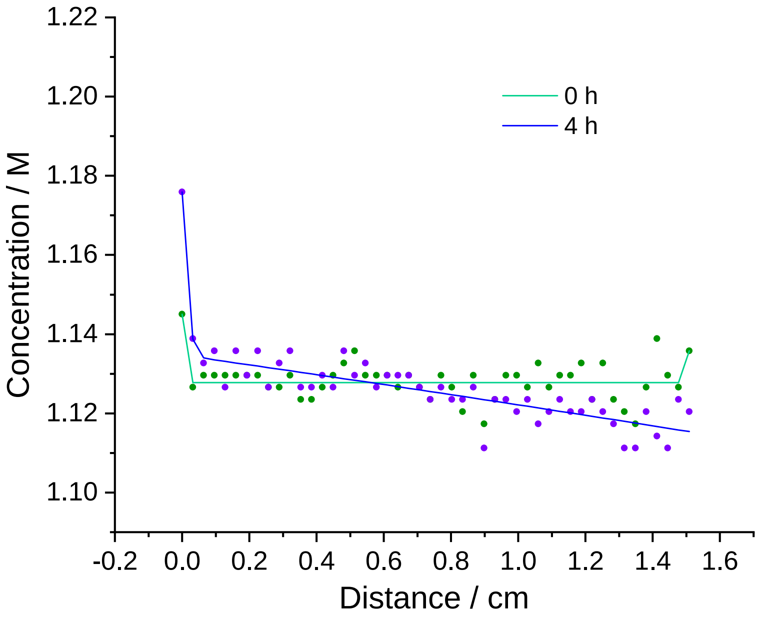

Supporting Figure 3: *Lithium concentration gradient between two stainless steel pistons prior to application of current. A smaller gradient forms compared to using lithium metal electrodes.*

2.4 Solvation Number

The Li^{+} solvation number was estimated by calculating the fraction of bound FSI species present using the areas of the fitted 1215.5 cm^{-1} and 1225 cm^{-1} peaks ($Area_{b(ratio)}$).[2]

$$a = \frac{A_b}{A_f + A_b} \quad (3)$$

Also, the fraction of $[Li^+]/[FSI^-]$ was calculated to understand how much Li^+ there is vs. FSI^-.

$$b = \frac{[Li^+]}{[FSI^-]} \quad (4)$$

If one assumes Li^+ is solvated:

$$SolvationNo. = \frac{a}{b} \quad (5)$$

From the fitting of the 1215.5 cm^{-1} and 1225 cm^{-1} peaks, with FWHM of 10, the estimated solvation number of Li^+ was ~2, independent of concentration. The deviation at low concentrations we attribute as a error in the fitting of a small bound-FSI peak.

Supplementary Table 2: *Solvation Number of Li^+*

$\textbf{Concentration}_{molality}$	$\textbf{Concentration}_{molarity}$	$\textbf{Area}_{boundFSI}$	$\textbf{Area}_{freeFSI}$	**Solvation Number**
0.05	0.066	0.19	9.90	1.25
0.10	0.13	0.69	8.91	2.41
0.25	0.32	1.68	9.87	2.03
0.50	0.63	3.60	9.47	2.06
0.75	0.91	5.60	9.38	1.99
0.90	1.08	6.81	9.12	1.97
1.00	1.19	7.26	9.17	1.88
1.25	1.44	8.16	8.82	1.87

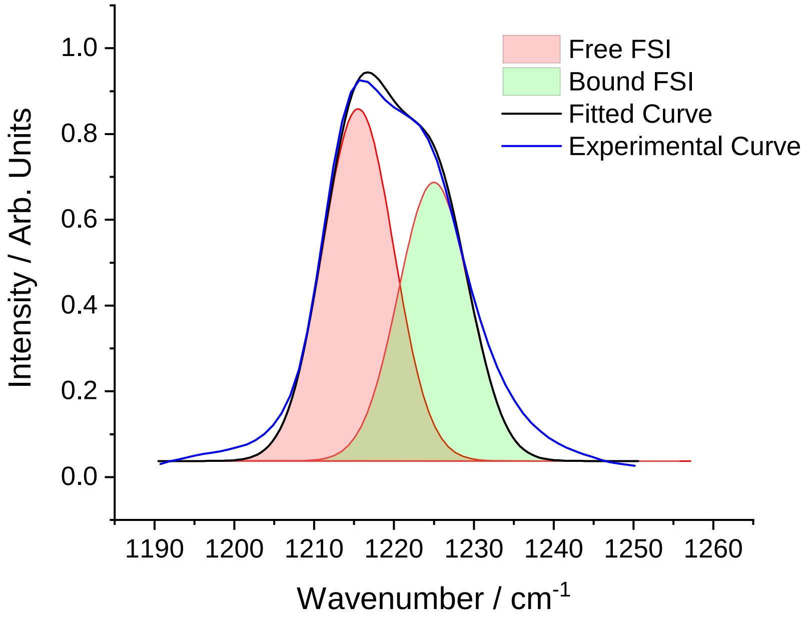

Supporting Figure 4: *Example fitting of 1220 cm^{-1} peak of 1 m LiFSI in Pyr$_{1,3}$FSI*

2.5 pfg-NMR

It is generally estimated D_{salt} is the harmonic mean of each ion's diffusion coefficient in solution from pfg-NMR:[3]

$$D_{salt} = \frac{c_T}{\frac{c_{Li^+}}{D_{Li^+}} + \frac{c_{FSI^-}}{D_{FSI^-}} + \frac{c_{Pyr^+}}{D_{Pyr^+}}} \tag{6}$$

Where c_T is the concentration total, $D_{Li^+} = 1.42 \times 10^{-11}$, $D_{FSI^-} = 1.88 \times 10^{-11}$, $D_{Pyr^+} = 1.76 \times 10^{-11}$ m^{2}s^{-1}. D_{salt} was calculated as 1.77×10^{-11} m^{2}s^{-1}.

Supporting References

(1) Lamb, H., *Hydrodynamics*, 4th; Cambridge University Press: 1916.

(2) Fujii, K.; Hamano, H.; Doi, H.; Song, X.; Tsuzuki, S.; Hayamizu, K.; Seki, S.; Kameda, Y.; Dokko, K.; Watanabe, M.; Umebayashi, Y. Unusual Li^+ Ion Solvation Structure in Bis(fluorosulfonyl)amide Based Ionic Liquid. *J. Phys. Chem. C* **2013**, *117*, 19314–19324.

(3) Bazak, J. D.; Soc, J. E.; Bazak, J. D.; Allen, J. P.; Krachkovskiy, S. A.; Goward, G. R. Mapping of Lithium-Ion Battery Electrolyte Transport Properties and Limiting Currents with In Situ MRI Mapping of Lithium-Ion Battery Electrolyte Transport Properties and Limiting Currents with In Situ MRI. *J. Electrochem. Soc.* **2020**, *167*, 140518.
