# Supplementary material for: Insights into the Transport and Thermodynamic Properties of a Bis(fluorosulfonyl)imide-Based Ionic Liquid Electrolyte for Battery Applications
Source: J Phys Chem Lett. 2022 Feb 16;13(7):1734–41. doi: 10.1021/acs.jpclett.1c04246 (PMC9084600; doi:10.1021/acs.jpclett.1c04246)
Supplement: Supplementary file 2 — jz1c04246_si_002.pdf [file jz1c04246_si_002.pdf]

jz-2021-04246j.R1

Name: Peer Review Information for "Insights into the Transport and Thermodynamic Properties of a Bis(fluorosulfonyl)imide-based Ionic Liquid Electrolyte for Battery Applications"

First Round of Reviewer Comments

Reviewer: 1

This paper demonstrates that concentration gradients in LiFSI-doped Pyr13FSI ILEs, when Li | the ILE | Li cell are polarized, can be visualized by operando Raman micro-spectroscopy for the first time. From the results, important transport properties in the ILEs, such as apparent diffusion coefficient, lithium transference number, and thermodynamic factor in addition to ionic conductivity and charge transfer resistance in contact with metallic Li by EIS.

This is a nice piece of work, which offered a new experimental methodology to reveal complex transport phenomena in ion-concentrated systems where correlated ionic motion is not negligible. This reviewer encourages the authors to expand this method to concentrated electrolyte solutions and polymer electrolytes. I have no major comments on this paper and recommend its publication in JPCL. The following is a small question that should be addressed before publication.

Page 9, bottom: "The magnitude of the diffusivities would suggest transport is occurring via ion-hopping mechanism as opposed to bulk motion of the aggregates identified in the previous sections."

Page 7, line 6: "Using Stokes Low we estimated the size of aggregates to be 3-8 μm; larger than others hypothesized."

This reviewer cannot understand the correlation between the above two statements.

Reviewer: 2

Comments to the Author

Report for JPCL:
Insights into the Transport and Thermodynamic Properties of an FSI-based Ionic Liquid Electrolyte

by Jack Fawdon, Gregory J. Rees, Fabio La Mantia and Mauro Pasta

The manuscript by Fawdon et al. submitted to JPCL is a revision of a manuscript sent earlier to JOURNAL. I am referring here to the comments given in my report on the previous manuscript (see also copied below), though due to the manuscript transfer no author reply is available.

As stated in that report, the Raman approach is interesting and timely, but was published before and novelty of the findings of the current study on another material needed to be pointed out more clearly. The authors have done so already in the title by moving the focus from the technique to the material. Similarly, the scope described in the introduction is focused more on the properties of the material, in particular showing the first concentration profiles for an IL electrolyte.

Only the conclusions still describe rather vaguely 'particularly valuable findings. These findings need to be clearly mentioned, in particular since any findings about the structure are still uncertain, see also issue below with large network-like aggregates contradicting the diffusion coefficients.

I rate the manuscript publishable after major revision.

Specific issues:

- Definitions of quantities and acronyms have been added. Before equation 1, please add b,d and a,c as diffusion length and interfacial gradient values.

- Definitions and explanations on page 2, lower paragraph, are still misleading and partially wrong: Line 37: "The most popular method for determining tLi+ in ILEs is via (electrophoretic) pulsed-field gradient nuclear magnetic resonance (pfg-(e)NMR) studies, and explicitly measures selfdiffusion coefficient of each ion in solution (Di) and subsequently calculates tLi+(NMR) by estimating the fraction of current carried by Li+ by knowing the product of Di and ci of each component (partial conductivity)." Note that in standard pfg-NMR diffusion coefficients are measured as the self-diffusion coefficient Di (from which transport numbers can be CALCULATED or transference number ESTIMATED under assumption of the Nernst-Einstein equation), whereas in eNMR electrophoretic mobilities are measured and transference number CALCULATED. This sentence mixes both methods, such that it remains unclear whether „calculates tLi+ by estimating…" refers to an estimate using the Nernst-Einstein equation, or whether it refers to a calculation from mobilities from eNMR. This part should be worked out more clearly with reference to the procedures established in literature.

- Page 3, line 7: "the restricted diffusion method": Which method is meant by this? Please elaborate on the method and give a reference. In this way of presentation the definition of Dapp is still not clear. A proper definition is absolutely needed! As presented here, it can easily be mixed up with averages over an ion species in different clusters from diffusion measurements as sometimes used in pfg-NMR, but here it seems to be rather an average salt diffusion coefficient, judging from the later context (page 9). In case of the latter, consider renaming it to Dsalt, to be consistent with electrochemical literature.

- This previous issue is not resolved: Application of Stokes law, page 7, line 13: The aggregate sizes are too large to agree to sizes extracted from the diffusion coefficient. It does not seem realistic that such large aggregates are the transport-relevant species. Is the kinetics

of gradient formation probably different from a sedimentation? Or is there something wrong with the diffusion coefficients in table 1?

- Page 6, line 5 sentence occurs twice

- Why does Fig. 5 show 2 data points per concentration? Why do some of them not agree within error?

Previous Report for JOURNAL:

Insights into Ionic Liquid Electrolyte Transport and Structure via Operando Raman Microspectroscopy

by Jack Fawdon, Gregory J. Rees, Fabio La Mantia and Mauro Pasta

The manuscript by Fawdon et al. applies the rather novel method of Raman microspectroscopy to a salt-in-ionic liquid electrolyte (ILE) system. The electrolyte is LiFSI in Pyr13FSI at three different concentrations. The authors use a calibration of the Li local concentration on a FSI Raman band and can thus evaluate Li concentration profiles at different times, i.e. cLi(x,t) obtained under constant current conditions. Fitting such profiles, in particular the concentration gradients at the plating and stripping side, respectively, they obtain a range of thermodynamic and transport parameters, such as the apparent diffusion coefficient and the Li transference number tLi, which are compared to results from other methods (PFG NMR). Further parameters obtained are the thermodynamic factor, conductivity and charge transfer resistance.

Some interesting aspects of the ILE behavior are revealed from these data, for example an asymmetry of the conc gradients due to Li-anion cluster formation and a gravimetrically driven sedimentation of Li-anion clusters to the plating side.

While this method – being based on time and position-dependent Raman spectra - is an interesting approach, I do not see a sufficiently novel claim which would justify publication in JOURNAL. The method and the same analysis have recently been published (Ref 20, Nature Comm.), where the authors used LiFSI in tetraglyme to establish the principle of this method. The present manuscript is a follow-up of this work, where LiFSI in Pyr13FSI is used. Most of the parameters extracted from the analysis are compared to results from other methods, which is nice to demonstrate the validity of the Raman approach, however, I am missing a clear statement of the novelty in terms of the results achieved on the particular system. Both abstract and conclusions only describe rather vaguely ‘insights into the bulk structure’ and ‘particularly valuable findings’. These insights and findings need to be clearly pointed out to provide a novel claim.

In summary, I rate the manuscript worth publishing in a more specialized Journal, such as J. Phys. Chem., or a electrochemical Journal, as it certainly presents novel data on the particular ILE system. A revision should point out the novelty more clearly. In addition, the presentation of the analysis and data needs clarification with respect to several issues, pointed out below.

Specific issues:

- Several quantities and acronyms lack definition. They all should be defined upon their first occurrence: thermodynamic factor on page 3, line 3; quantities in equ. (1)-(3) are not defined in the text; parameters in caption of Fig 1, Rct for example is only explained on page 10, line 46; PEIS;…
- Especially important is to use exact definitions for Dapp and tLi throughout the manuscript. A distinction between values obtained by different methods needs to be made, note that tLi has different definitions and reaches different values when obtained from anion-blocking conditions (tLiabc), PFG-NMR (tLiDiff), which both differ from the definition by partial conductivities, which is obtained by electrophoretic NMR. This should be clarified in the introduction, lower half of page 2.
- More details of the experimental setup (electrodes used, dimensions of the cell etc.) should be given instead of referring to the previous paper.
- The text is in some passages hard to understand without reading Ref 20, more detail and more clarity should be provided. For example, page 4/5: 'we checked the mass balance by integrating each concentration profile over the 36 hour experiment': I suppose this is not an integration of time (36 h). but rather over position for each species concentration separately?
- Application of Stokes law, bottom of page 5: The aggregate sizes are too large to agree to sizes extracted from the PFG-diffusion coefficients. It does not seem realistic that such large aggregates are the transport-relevant species. Is the kinetics of gradient formation probably different from a sedimentation? Or is there something wrong with the diffusion coefficients in table 1?
- Page 2, line 29: Instead of '3 or 4 ions' probably ion species are meant.
- In several points the manuscript is too brief and needs extended explanations, for example 1) page 10, line 40: What are Richter findings, exact value? 2) page 5, line 33 agreeing well: please show evidence in numbers; 3) page 10, line 44 'measure ' the ionic conductivity: It is obtained froma fit, isn't it?
- Page 10, line 48: 'Haven ratio': should probably read 'inverse Haven ratio'
- Ideas of a percolating network in ILE are not new, see recent simulations by Molinari and Kozinski. Please point out which conclusions from the data are novel.
- Why does Fig. 5 show 2 data points per concentration? Why do they not agree within error?

Author's Response to Peer Review Comments:

Insights into the Transport and Thermodynamic Properties of a Bis(fluorosulfonyl-based Ionic Liquid Electrolyte for Battery Applications

Jack Fawdon[a], Gregory Rees[a,b], Fabio La Mantia[b], Mauro Pasta[*a,b]

[a]Department of Materials, University of Oxford, Parks Rd, Oxford, OX1 3PH, U.K.
[b]The Faraday Institution Quad One, Harwell Science and Innovation Campus, Didcot, OX11 0RA, U.K.
[c]Universität Bremen, Energiespeicher-und Energiewandlersysteme, Bibliotechkstra$\boldsymbol{\beta}$e 1, Bremen, 28359, Germany

*E- mail: mauro.pasta@materials.ox.ac.uk

We'd like to thank all the Reviewers for their constructive and insightful comments. Changes implemented to the original document are reported in blue and highlighted in yellow for the direct quote to the revised manuscript.

Non-scientific changes requested by senior editor:

Please make the following non-scientific changes:

1) Title: Using acronyms in title is discouraged. Please spell out all acronyms in the title of the manuscript and Supporting Information.

The title has been changed:

Insights into the Transport and Thermodynamic Properties of a Bis(fluorosulfonyl)imide-based Ionic Liquid Electrolyte for Battery Applications

2) Author Lists: Please add a full header at top of the first page of the manuscript file, which includes: title, full author list, and author affiliations (exactly as they appear in the SI, if one is supplied). Please format your author and affiliation information according to the guidelines in this link: https://pubsapp.acs.org/paragonplus/submission/author-address-information.pdf. (Please add postal codes to all affiliation addresses.)

A postal code has been added to the Faraday Institution affiliation.

3) Supporting Information: Please number pages in the following format: "S1, S2…"

Page numbers have been amended as requested.

*4) References: In both the main file and the supporting information, fix the style of all references to use JPCL formatting (check all references carefully). ***JPC Letters reference formatting requires that journal references should contain: () around numbers, author names, article title (titles entirely in title case or entirely in lower case), abbreviated journal title (italicized), year (bolded), volume (italicized), and pages (first-last). Book references should contain author names, book title (in the same pattern), publisher, city, and year.*

Formatting of the references has been amended in both the main text and supporting information.

5) TOC Graphic: Provide a TOC image per journal guidelines (2 in x 2 in; on the same page as the abstract) with the heading "TOC Graphic" above the graphic. The graphic should be in the form of a structure, graph, drawing, photograph, or scheme—or a combination. Non-scientific cartoon-like images or caricatures are discouraged. https://pubsapp.acs.org/paragonplus/submission/toc_abstract_graphics_guidelines.pdf

We also require a graphic for the "Table of Contents" with all submissions. We ask that you include a graphic immediately after the Abstract under the header "TOC Graphic." Instructions are available at http://pubs.acs.org/paragonplus/submission/jpchax/jpclcd_authguide.pdf .

TOC Graphic:

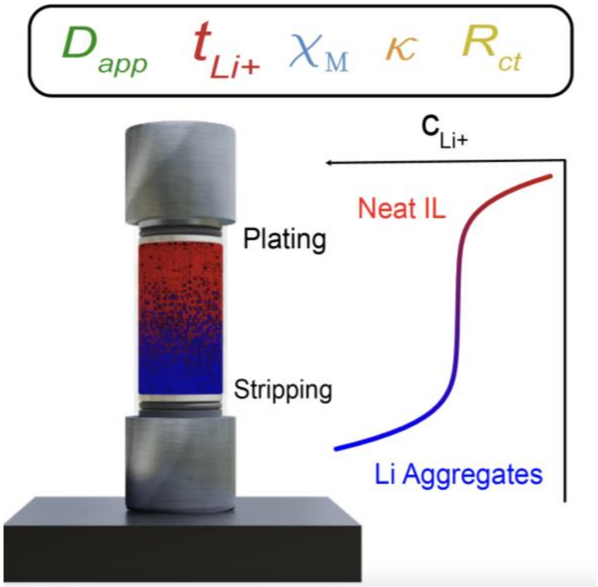

Response to Reviewers' Comments

Reviewer: 1

This paper demonstrates that concentration gradients in LiFSI-doped Pyr13FSI ILEs, when Li | the ILE | Li cell are polarized, can be visualized by operando Raman micro-spectroscopy for the first time. From the results, important transport properties in the ILEs, such as apparent diffusion coefficient, lithium transference number, and thermodynamic factor in addition to ionic conductivity and charge transfer resistance in contact with metallic Li by EIS.

This is a nice piece of work, which offered a new experimental methodology to reveal complex transport phenomena in ion-concentrated systems where correlated ionic motion is not negligible. This reviewer encourages the authors to expand this method to concentrated electrolyte solutions and polymer electrolytes. I have no major comments on this paper and recommend its publication in JPCL.

We would like to thank the Reviewer for their positive comments, highlighting our work's significance, and the complexity of the system it characterises.

The following is a small question that should be addressed before publication.
Page 9, bottom: "The magnitude of the diffusivities would suggest transport is occurring via ionhopping mechanism as opposed to bulk motion of the aggregates identified in the previous sections."

We would like to apologise for the confusion if these statements appeared to correlate.

The first statement (Page 9, bottom) was stating that because the pfg-NMR D_{app} value (which does not include bulk motion due to gravity) was the same as the diffusivity value measured via the movement of the diffusion length (which does include bulk motion due to gravity), it was deduced that the vast majority of the diffusion measured via the movement of diffusion length was due to diffusion via an ion-hopping method and not due to the sedimentation of large aggregates that were identified in the previous section. To avoid confusion, this statement has been changed to:

Page 10, Lines 11-14: "The magnitude of the diffusivities would suggest transport is occurring primarily via an ion-hopping mechanism as opposed to sedimentation of the aggregates identified in the previous sections. However, sedimentation is proposed to be the reason for the asymmetric concentration gradient that was visualised."

Page 7, line 6: "Using Stokes Low we estimated the size of aggregates to be 3-8 m; larger than others hypothesized."
This reviewer cannot understand the correlation between the above two statements.

The second statement (Page 7, line 6) was estimating the size of the aggregates from Stokes Law. When "...others hypothesized." was stated, this was referring to other studies that have estimated size experimentally (via X-ray or NMR techniques) or simulations (via MD).

For clarity, references have been added to this second statement:

Page 7, Line 10: “Using Stokes’ Law, we estimated the size of aggregates to be 3-8 um; larger than others hypothesized.[31,32]”

(31) Russina, O.; Lo Celso, F.; Di Michiel, M.; Passerini, S.; Appetecchi, G. B.; Castiglione, F.; Mele, A.; Caminiti, R.; Triolo, A. Mesoscopic structural organization in triphilic room temperature ionic liquids. Faraday Discuss. 2013, 167, 499–513.

(32) Pontoni, D.; Haddad, J.; Di Michiel, M.; Deutsch, M. Self-segregated nanostructure in room temperature ionic liquids. Soft Matter 2017, 13, 6947–6955.

Reviewer: 2

The manuscript by Fawdon et al. submitted to JPCL is a revision of a manuscript sent earlier to JOURNAL. I am referring here to the comments given in my report on the previous manuscript (see also copied below), though due to the manuscript transfer no author reply is available.

As stated in that report, the Raman approach is interesting and timely, but was published before and novelty of the findings of the current study on another material needed to be pointed out more clearly. The authors have done so already in the title by moving the focus from the technique to the material. Similarly, the scope described in the introduction is focused more on the properties of the material, in particular showing the first concentration profiles for an IL electrolyte.

Only the conclusions still describe rather vaguely ‘particularly valuable findings. These findings need to be clearly mentioned, in particular since any findings about the structure are still uncertain, see also issue below with large network-like aggregates contradicting the diffusion coefficients.

I rate the manuscript publishable after major revision.

We would like to thank the Reviewer for their positive comments regarding our experimental methodology and for noting the changes that were made after our submission to JOURNAL. We appreciate the opportunity to improve our manuscript by addressing the questions they have. Each of these specific queries in the JOURNAL review process have been addressed, unless Reviewer 2 otherwise stated in the comments above.

The Reviewer has requested for us to be clearer about our conclusions and findings within the paper. To be explicit, this is the first time:

1) The concentration gradient of an ionic liquid electrolyte has been visualised.
2) Four key electrolyte properties (D_{app}, t_{Li+}, κ and χ_M) of an ILE have been measured in one study.

3) Gravimetric sedimentation of large clusters has been detected in ionic liquid electrolyte solutions.

Each of these findings is of value to those interested in ionic liquid electrolytes and the wider battery community.

The conclusion has been updated to clearly describe the key novelties and findings of our work:

Page 14, Lines 8-19: "In summary, by combining spectroscopic and electrochemical techniques with concentration visualisation we have presented particularly valuable findings not yet reported in the ILE literature. Specifically, the Li^+ concentration gradient in ILEs has been visualised for the first time, along with the isolation of key transport and thermodynamic properties. With ILEs' main weakness being their transport properties, understanding fully the origin of this is paramount for their continuing development. Moreover, this is the first time a thermodynamic understanding of promising battery ILEs has been measured through χ_M. Additionally, the sedimentation of clustered aggregates have been detected, which have not yet been experimentally measured in the academic literature until now. ."

Specific issues:
- Definitions of quantities and acronyms have been added. Before equation 1, please add b,d and a,c as diffusion length and interfacial gradient values.

The following statement has been amended to include b,d and a,c definitions (page 8 and 9)

Page 8, bottom: " *b* and *d* are equal to L_d, defined as the diffusion length, and *a* and *c* are equal to $dc_{Li+}/dz|_{z=0,L}$, which is the interfacial concentration gradient at each electrode surface, *z=0, L*."

- Definitions and explanations on page 2, lower paragraph, are still misleading and partially wrong: Line 37: "The most popular method for determining tLi+ in ILEs is via (electrophoretic) pulsed-field gradient nuclear magnetic resonance (pfg-(e)NMR) studies, and explicitly measures selfdiffusion coefficient of each ion in solution (Di) and subsequently calculates tLi+(NMR) by estimating the fraction of current carried by Li+ by knowing the product of Di and ci of each component (partial conductivity)." Note that in standard pfg-NMR diffusion coefficients are measured as the self-diffusion coefficient Di (from which transport numbers can be CALCULATED or transference number ESTIMATED under assumption of the Nernst-Einstein equation), whereas in eNMR electrophoretic mobilities are measured and transference number CALCULATED. This sentence mixes both methods, such that it remains unclear whether „calculates tLi+ by estimating…" refers to an estimate using the Nernst-Einstein equation, or whether it refers to a calculation from mobilities from eNMR. This part should be worked out more clearly with reference to the procedures established in literature.

We thank the Reviewer for highlighting the difference between electrophoretic and standard pfg-NMR methods for *estimating* and *calculating* the transference number.

Changes were made to the manuscript to clarify these differences:

Page 3, Lines 1-5: "Without an electric field (i.e. pfg-NMR), the transference number can be estimated by calculating the fraction of current carried by Li^+ using the product of D_i and c_i of each component (partial conductivity). By applying an electric field, pfg-eNMR can measure the mobility of the ions in solution, and subsequently isolate the true transference number."

- Page 3, line 7: "the restricted diffusion method": Which method is meant by this? Please elaborate on the method and give a reference. In this way of presentation the definition of Dapp is still not clear. A proper definition is absolutely needed! As presented here, it can easily be mixed up with averages over an ion species in different clusters from diffusion measurements as sometimes used in pfg-NMR, but here it seems to be rather an average salt diffusion coefficient, judging from the later context (page 9). In case of the latter, consider renaming it to Dsalt, to be consistent with electrochemical literature.

"The restricted diffusion method" is a common method for measuring the "apparent" or "effective" diffusion coefficient (used interchangeably, D_{app} and D_{eff} respectively), measured by groups such as Newman, Monroe, Gasteiger, Reimers and Lindeberg as referenced below. Using a 2-electrode cell of known interelectrode distance (L), and following the relaxation from an arbitrarily formed concentration gradient, the semi-log of the OCV decay can be followed with time. The ln(OCV) curve eventually becomes linear after enough time has elapsed, and the resulting slope is proportional to D_{app}.

$$lim(t \rightarrow \infty)\frac{dln(OCV)}{dt} = \frac{-\pi^2 D_{app}}{L^2}$$

This is equivalent to the movement of the diffusion length as monitored by concentration gradient visualisation in our methodology. This is why D_{app} is continued to be used in our manuscript.

Additions have been made to include a brief description of the "restricted diffusion method". Readers are invited to read the referenced publications that explicitly utilise the restricted diffusion method.

Page X Line X, "…nor has the "apparent" diffusion coefficient D_{app} been measured via the restricted diffusion method; a common method that measures D_{app} by monitoring the semi-log decay of open circuit voltage (OCV) over time, after an arbitrarily formed concentration gradient has been formed.[16-20] "

References:

(16) Hou, T.: Monroe, C.W. Composition-dependent thermodynamic and mass-transport characterization of lithium hexafluorophosphate in propylene carbonate. Electrochim. Acta 2020, 332, 135085.

(17) Ma, Y.; Doyle, M.; Fuller, T. F.; Doeff, M. M.; De Jonghe, L. C.; Newman, J. The Measurement of a Complete Set of Transport Properties for a Concentrated Solid Polymer Electrolyte Solution. J. Electrochem. Soc. 1995, 142, 1859–1868.

(18) Valoen, L. O.; Reimers, J. N. Transport Properties of $LiPF_6$-Based LiIon Battery Electrolytes. J. Electrochem. Soc. 2005, 152, A882.

(19) Nyman, A.; Behm, M.; Lindbergh, G. Electrochemical characterisation and modelling 18 of the mass transport phenomena in $LiPF_6$ -EC-EMC electrolyte. Electrochim. Acta 2008, 53, 6356–6365.

(20) Ehrl, A.; Landesfeind, J.; Wall, W. A.; Gasteiger, H. A. Determination of Transport Parameters in Liquid Binary Lithium Ion Battery Electrolytes: Part I. Diffusion Coefficient. J. Electrochem. Soc. 2017, 164, A826–A836.

We thank the Reviewer for the suggestion of changing the average salt diffusion coefficient calculated via pfg-NMR to be renamed to D_{salt}. We have thus changed each of these mentioned variable names accordingly:

Main Text: Page 9, Lines 17 and 20

Supporting Information: Page S7, Line 1 and 4

- This previous issue is not resolved: Application of Stokes law, page 7, line 13: The aggregate sizes are too large to agree to sizes extracted from the diffusion coefficient. It does not seem realistic that such large aggregates are the transport-relevant species. Is the kinetics of gradient formation probably different from a sedimentation? Or is there something wrong with the diffusion coefficients in table 1?

We apologise to the Reviewer for the confusion here. Stokes' law is utilised prior to any current flow, so it does not reference the diffusion coefficient. When current is passed, we do not state that transport is primarily based on sedimentation. However, we do propose that sedimentation is the reason for the asymmetry of the concentration gradient and does potentially contribute partly to the formation of the gradient while current is flowing. In the original manuscript we mention on page 9:

"The magnitude of the diffusivities would suggest transport is occurring via an ion-hopping mechanism as opposed to bulk motion of the aggregates identified in the previous sections."

To make this statement clearer, "bulk motion" has been changed to "sedimentation". And "primarily" is included so it does not discount the potential contribution of sedimentation:

Page 10, Lines 11-14: "The magnitude of the diffusivities would suggest transport is occurring primarily via an ion-hopping mechanism as opposed to sedimentation of the aggregates identified in the previous sections. However, sedimentation is proposed to be the reason for the asymmetric concentration gradient that were visualised."

- Page 6, line 5 sentence occurs twice

We thank the Reviewer for noticing this error. The first occurrence of the repeated sentence has been removed.

- Why does Fig. 5 show 2 data points per concentration? Why do some of them not agree within error?

For each concentration we performed two measurements, as stated on page 12, line 16. The standard error was calculated during the fitting procedure for each measurement, specifically for the diffusion length and the interfacial concentration gradient. The error between concentrations is therefore dependent on the fitting parameters and points gathered when measuring the concentration during the line scan. Due to there being an asymmetric gradient, an inverse-weighted mean based on the error of each side, along with its standard deviation, was calculated. For clarity, an additional sentence is added to Fig. 5's caption:

Figure 5 "...The error bars represent the standard deviation of the inverse-weighted mean of the stripping and plating sides from the fitting of the asymmetric gradient."

The differences in value at the same concentration, between two measurements, also represents experimental error within our setup that was difficult to control. For example, there were slight variations in temperature that could be the cause of this. Additionally, for sufficient spectral resolution, we had to go beyond the critical current of dendrite formation. The random nature of dendrite growth led to some experimental inconsistencies, which could also explain the difference in error values in Fig. 5. An additional statement is added to Fig. 5 caption:

Figure 5. "Experimental inconsistencies such as dendrite formation and small temperature variations explain some differences between the calculated property values for repeated experiments."
